# Normobaric hypoxia shows enhanced FOXO1 signaling in obese mouse *gastrocnemius* muscle linked to metabolism and muscle structure and neuromuscular innervation

**DOI:** 10.1007/s00424-023-02854-4

**Published:** 2023-09-01

**Authors:** Jingyi Song, Loes P. M. Duivenvoorde, Sander Grefte, Ondrej Kuda, Felipe Martínez-Ramírez, Inge van der Stelt, Dimitra Mastorakou, Evert M. van Schothorst, Jaap Keijer

**Affiliations:** 1 Human and Animal Physiology, Wageningen University, Wageningen, The Netherlands; 2 Laboratory of Metabolism of Bioactive Lipids, Institute of Physiology, Czech Academy of Sciences, 14220 Prague 4, Czech Republic

**Keywords:** Hypoxia, Skeletal muscle, FOXO, Mitochondria, Metabolism, Neuromuscular junction

## Abstract

Skeletal muscle relies on mitochondria for sustainable ATP production, which may be impacted by reduced oxygen availability (hypoxia). Compared with long-term hypoxia, the mechanistic *in vivo* response to acute hypoxia remains elusive. Therefore, we aimed to provide an integrated description of the *Musculus gastrocnemius* response to acute hypoxia. Fasted male C57BL/6JOlaHsd mice, fed a 40en% fat diet for six weeks, were exposed to 12% O_2_ normobaric hypoxia or normoxia (20.9% O_2_) for six hours (*n* = 12 per group). Whole-body energy metabolism and the transcriptome response of the *M. gastrocnemius* were analyzed and confirmed by acylcarnitine determination and Q-PCR. At the whole-body level, six hours of hypoxia reduced energy expenditure, increased blood glucose and tended to decreased the respiratory exchange ratio (RER). Whole-genome transcriptome analysis revealed upregulation of forkhead box-O (FOXO) signalling, including an increased expression of tribbles pseudokinase 3 (*Trib3*). *Trib3* positively correlated with blood glucose levels. Upregulated carnitine palmitoyltransferase 1A negatively correlated with the RER, but the significantly increased in tissue C14-1, C16-0 and C18-1 acylcarnitines supported that β-oxidation was not regulated. The hypoxia-induced FOXO activation could also be connected to altered gene expression related to fiber-type switching, extracellular matrix remodeling, muscle differentiation and neuromuscular junction denervation. Our results suggest that a six-hour exposure of obese mice to 12% O_2_ normobaric hypoxia impacts *M. gastrocnemius* via FOXO1, initiating alterations that may contribute to muscle remodeling of which denervation is novel and warrants further investigation. The findings support an early role of hypoxia in tissue alterations in hypoxia-associated conditions such as aging and obesity.

**Supplementary Information:**

The online version contains supplementary material available at 10.1007/s00424-023-02854-4.

## Introduction

Oxygen is vital for mammalian life due to its role in cellular ATP production, mainly in the mitochondria via oxidative phosphorylation. Mitochondria consume over 90% of oxygen in the body of which over 80% is used for ATP production [82]. Next to ATP production, mitochondria perform other essential functions such as cellular calcium handling, metabolic coordination, programmed cell death, regulating redox balance and signaling, and various biosynthesis reactions, including heme and iron-sulfur cluster biosynthesis [14, 39]. In doing this, mitochondria are highly adaptive, allowing them to align these processes to cellular needs. Skeletal muscle, the largest tissue in the body, contributes to approximately 21% of oxygen consumption in rest [102]. Mitochondrial adaptivity is also evident in skeletal muscle, where oxygen demand increases substantially during activity, with a ten-fold increase in oxygen dependent ATP production during exercise [38]. Therefore, it is not surprising that reduced tissue oxygen availability, also called hypoxia, impacts on skeletal muscle mitochondria.

Hypoxia occurs in organisms at high-altitude and in peripheral tissues when tissue oxygen delivery is impaired, for example, due to diseases such as pulmonary disease, cancer and obesity [74]. Additionally, pulmonary dysfunction in COVID-19 infection is characterized by hypoxia [2]. The consequential reduced blood oxygen level of COVID-19 results in dysfunction of the diaphragm muscle and a reduction in limb skeletal muscle mass [56, 80]. Generally, conditions of long-term hypoxia can cause loss of body mass (fat and lean mass) in human [50], and enhanced lipid catabolism in obese mice [93]. In addition to long-term hypoxia, acute hypoxia can occur, for example, during sleep apnea [71], on air flights [51] or with short bouts of moderate exercise, especially in overweight, older and pregnant individuals [63]. Long-term hypoxia comes with various long-term tissue adaptations, but whether skeletal muscle also adapts to acute hypoxia is poorly investigated.

Skeletal muscle mitochondria can use different substrates to produce ATP, such as pyruvate, fatty acids, amino acids and ketone bodies. During short bouts of intensive exercise, ATP can also be derived from glycolysis in the cytosol in an oxygen-independent manner [5]. Thus, skeletal muscle possesses the metabolic flexibility to maintain cellular energetics, also during hypoxia. Indeed during short (1–14 days), medium (14–42 days) and long-term hypoxia (> 42 days), oxidative phosphorylation is attenuated whilst glucose uptake is maintained or increased [40]. At the same time pyruvate dehydrogenase kinase (PDK) is upregulated, inhibiting pyruvate dehydrogenase and reducing the entry of pyruvate into the TCA cycle. In addition, hypoxia lowered mitochondrial density especially in the subsarcolemmal population of the skeletal muscle mitochondria [60, 73], and switched complex IV subunits to improve electron transfer efficiency and oxygen usage for oxidative ATP production in cells [30]. Together, these data suggest that ATP synthesis in the hypoxic environment in muscle is optimized by an increase in glycolysis to compensate the downregulation of oxidative metabolism and/or an optimization of oxidative metabolism. In addition to these metabolic adaptations, skeletal muscle structure was also shown to be affected by hypoxia. In the *M. soleus,* intermittent hypoxia for 42 days resulted in increased type I and decreased type IIa fibers and the total cross sectional area and the size of neuromuscular junctions (NMJ) decreased, but these changes were not observed in the *M. gastrocnemius* [4].

Several transcription factors are involved in the adaptation of skeletal muscle to long-term hypoxia, of which the best known transcription factor is hypoxia-inducible factor (HIF)1A. HIF1A regulates the expression of a large number of target genes involved in restoring cellular energy homeostasis [87]. Additionally, the Forkhead Box-O (FOXO) family of transcription factors, of which FOXO1, FOXO3, FOXO4 and FOXO6 are all expressed in skeletal muscle [84]. FOXO has been reported to promote hypoxia tolerance in *Drosophila*, skeletal muscle of fish and hippocampus of mice [6, 18, 61]. Highly conserved across animals, FOXO is upregulated in skeletal muscle in energy-deprived states, such as fasting and severe diabetes, and modulates autophagy and energy homeostasis [84]. More specifically, during fasting FOXO1 reduced glycolysis, presumably via regulating PDK4 [7, 31]. In addition, FOXO seems to upregulate fatty acid oxidation by regulating the levels of lipoprotein lipase, fatty acid translocase, and adiponectin receptors [7, 47, 97]. This suggests that FOXO1 can act as a metabolic switch [29, 84]. Conversely, in hypoxic conditions, increased *Foxo3a* expression was related to reduced mitochondrial respiration and increased glycolysis suggesting a dual metabolic role [44]. FOXO1 also plays a role in skeletal muscle development. FOXO1 inhibits myoblast differentiation in the early phase, but stimulates myotube formation in the later phase of myogenesis. Increased expression of FOXO1 and FOXO3 *in vivo* resulted in decreased body size and skeletal muscle mass [12, 41, 54]. Together, this suggests a role of FOXOs in controlling both metabolic and structural adaptations in skeletal muscle to hypoxia.

Since most studies focused on long-term hypoxic exposure, the mechanism of skeletal muscle adaptation to acute hypoxia, representing sleep apnea [71], air flights [51] or short bouts of moderate exercise [63] remains elusive. An insight into acute *in vivo* responses to hypoxia will also help to understand the role of hypoxia in skeletal muscle adaption during aging and obesity, which are long-term conditions that are characterized by reduced tissue oxygen availability. Therefore, in this study, we aimed to better understand the *in vivo* effect of acute normobaric hypoxia on skeletal muscle. We studied the *M. gastrocnemius* as it is a major calf muscle that provides the force behind propulsion for walking, running, jumping and flexing the leg at the knee joint and the foot at the ankle joint [76]. *M. gastrocnemius* is composed of mixed fiber types with approximately 16% slow fibers [70] and is well suited to study the overall environmental responses on skeletal muscles. We exposed adult male C57BL/6JOlaHsd mice, fed a 40en% fat diet for six weeks, for six hours to 12% O_2_ normobaric hypoxia, similar to the ambient oxygen level of Pikes Peak (4302 m high). Six hours of hypoxia condition was chosen to allow us to examine the effects of this physiologically relevant acute hypoxia challenge at the transcriptome level. In addition, whole body energy expenditure and the respiratory exchange ratio (RER) were determined, as well as fasting blood glucose, serum insulin levels, and skeletal muscle acylcarnitines to functionally characterize muscle lipid metabolism.

## Materials and methods

### Experimental design

Twenty-four male C57BL/6JOlaHsd mice were purchased from Harlan Laboratories (Horst, The Netherlands). Mice arrived at ten weeks of age and were individually housed and maintained under environmentally controlled conditions (21 ± 1 °C, 12 h/12 h light–dark cycle, 50 ± 10% humidity) and had ad libitum access to food and water. During the first three weeks, mice adapted to the new environment and received the purified low-fat BIOCLAIMS standard diet that contains 10% energy from fat [37]. Thereafter, mice received a purified BIOCLAIMS diet for six weeks with 40% energy from fat, corresponding to the average fat percentage in the human diet in the Netherlands [28, 101]. Body weight and body composition were monitored weekly by EchoMRI Whole Body Composition Analyser (EchoMRI, Houston, FL, USA). Adiposity was calculated as (total fat mass / body weight) × 100%. For the study, the then 18 weeks old mice were randomly allocated to the experimental hypoxia (Hypox) group or the control normoxia (Norm) group (*n* = 12 per group) and housed in the indirect calorimetry (InCa) system (see below). After exposure to normobaric hypoxic (12% O_2_) or normoxic (20.9% O_2_) air during six hours, mice were immediately killed by decapitation. Six hours exposure to normobaric hypoxia was chosen because VO2 was stable (after an initial increase) after 5 h [27]. Blood was collected in Mini collect serum tubes (Greiner Bio-one, Longwood, FL, USA). Serum tubes were centrifuged at 3000 *g* for 10 min at 4 °C to obtain serum and then aliquoted and stored at -80 °C until analyses. Tissues were excised, snap frozen in liquid nitrogen and stored at -80 °C until analyses.

### Indirect calorimetry and hypoxia exposure

The InCa system (PhenoMaster system, TSE Systems, Bad Homburg, Germany) was used to measure whole-body energy metabolism and create a hypoxic environment as described above, which includes incorporation of a hypoxia pump (B-Cat, Tiel, The Netherlands) [27, 28]. In short, mice adapted in normoxia to the InCa system for 24 h. All feed was removed at the start of the dark phase, and mice received a restricted amount of feed (1.5 g) to ensure a fasting state at the start of the following light phase. One hour after the start of the light phase, oxygen concentration was decreased in each animal cage from 20.9% to 12% in the Hypox group and VO_2_ and VCO_2_ were recorded every 13 min for the following six hours. Mice in the Norm group were treated in the same manner, but remained under normoxic conditions (ambient air; 20.9% O_2_). Normobaric air pressure is standard in all situations.

### Blood measurements

After killing the mice, blood glucose concentration was measured in whole blood using a Freestyle blood glucose system (Abbott Diabetes Care, Hoofddorp, The Netherlands) according to the manufacturer’s instructions. Serum insulin concentration was measured with an Ultra-Sensitive Mouse Insulin ELISA Kit (Crystal Chem, Zaandam, The Netherlands) following the manufacturer’s instructions. Samples were tested in duplicate and averaged when coefficient of variation was less than 5%.

### RNA isolation and whole genome transcriptome analysis

The whole *M. gastrocnemius* was grinded for RNA isolation of which ten milligram was homogenized using the Tissue lyser II (Qiagen) for 3 min at 30 Hz, after which total RNA was isolated with the RNeasy Fibrous Tissue Mini Kit following the manufacturer’s instruction (Qiagen, Venlo, Netherlands). RNA concentration and integrity were measured by Nanodrop (IsoGen Life Science, Maarssen, Netherlands) and the Experion automated electrophoresis system (Bio-Rad).

For whole genome transcriptome analysis, 8 × 60 K Agilent whole-mouse genome microarrays (G4852A, Agilent Technologies Inc., Santa Clara, CA, USA) were used according to the manufacturer’s protocol with a few modifications as described previously [36]. All arrays were deposited in Gene Expression Omnibus (GEO) with accession ID: GSE228719. In total, 33,845 of the 59,305 probes on the array had a fluorescent signal twice above the background signal and were included for statistical analysis. Gene expression data was based on a two group comparison: 12 Hypox mice and 12 Norm mice. Statistical analyses of gene expression data were performed using GeneMaths XT version 2.12 (Applied Maths, Sint-Martens-Latem, Belgium), and P-values were calculated with the Student’s t-test based on Log2-normalized expression values. Transcripts with *P*-value < 0.05 were considered significantly regulated and used for further analysis.

Gene set enrichment analysis (GSEA) was performed in R version 4.1.0 with package clusterProfiler [107]. Gene sets with Benjamini–Hochberg adjusted *P*-value < 0.05 were considered as significantly enriched. With Gene Ontology (GO) gene set, GSEA was performed with all three aspects (biological process, cellular component and molecular function) and cellular component separately. The most significant 25 pathways were clustered together based on semantic similarities in GO description of the genes in each pathway, using the GoSEMSim package [108]. Kyoto Encyclopedia of Genes and Genomes (KEGG) [49], the mouse MitoCarta 3.0 gene set [81], and the MatrisomeDB gene set [89] were used as reference inventory for biological pathways, mitochondrial genes and extracellular matrix (ECM) genes respectively. For synaptic gene analysis, genes were first converted to matched human genes by BioMart on Ensembl [22, 53] after that an analysis with SynGO platform was performed [55]. Based on literature, a set specific of genes involved in NMJ was created [9, 26, 42, 43, 52, 68, 78].

### Quantitative reverse transcription polymerase chain reaction (Q-PCR)

cDNA synthesis and real-time Q-PCR were performed as described [27], using DEPP1 autophagy regulator (*Depp1*) and titin (*Ttn*) as target genes. Beta-2 microglobulin (*B2m*), calnexin (*Canx*), and ribosomal protein S15 (*Rps15*) were used as reference genes. Primers were designed by NCBI Primer BLAST. Sequences and product length of target and reference genes can be found in Table S1. Data were expressed as relative gene expression based on reference genes.

### Tissue acylcarnitine determination

*M. gastrocnemius* free carnitine and acylcarnitine levels were determined using an ultra-high performance liquid chromatography coupled with a mass spectrometry (UHPLC-MS) (UltiMate3000 RSLC, Thermo Scientific, Sunnyvale, CA, USA; QTRAP 5500, AB-Sciex, Framingham, MA, USA) as described previously [99]. A reference standard with labeled free (L-)carnitine and acylcarnitines (Chromsystems Instruments and Chemicals GmbH, Gräfelfing, Germany) was added to each sample for quantification. The identity of the metabolites was determined by retention time (t_R_) correlation. Moreover, the concentration of individual metabolites was calculated with the peak area of the metabolite of interest and the peak area of the corresponding standard.

### Statistics

The data were expressed as mean ± SD, geometric mean ± geometric SD for lognormal distribution data, and median (IQR) for non-normal data. All analyses were based on the data of 12 Hypox mice and 12 Norm mice; except for the measurement of physical activity which was based on 8 random selected mice per experimental group. Statistical analyses were performed using GraphPad Prism version 9.3.1 (Graphpad, San Diego, CA, USA), except for microarray data (see above). Data were checked for normality using the D’Agostino normality test. All normal data and lognormal data were analyzed by independent Student’s unpaired t-tests, except for free carnitine and acylcarnitines where Welch t-test was used. Non-normal data were analyzed with a Mann–Whitney test. *P*-values < 0.05 were considered statistically significant.

## Results

### Whole body effects of six hours hypoxia exposure

**Fig. 1:**
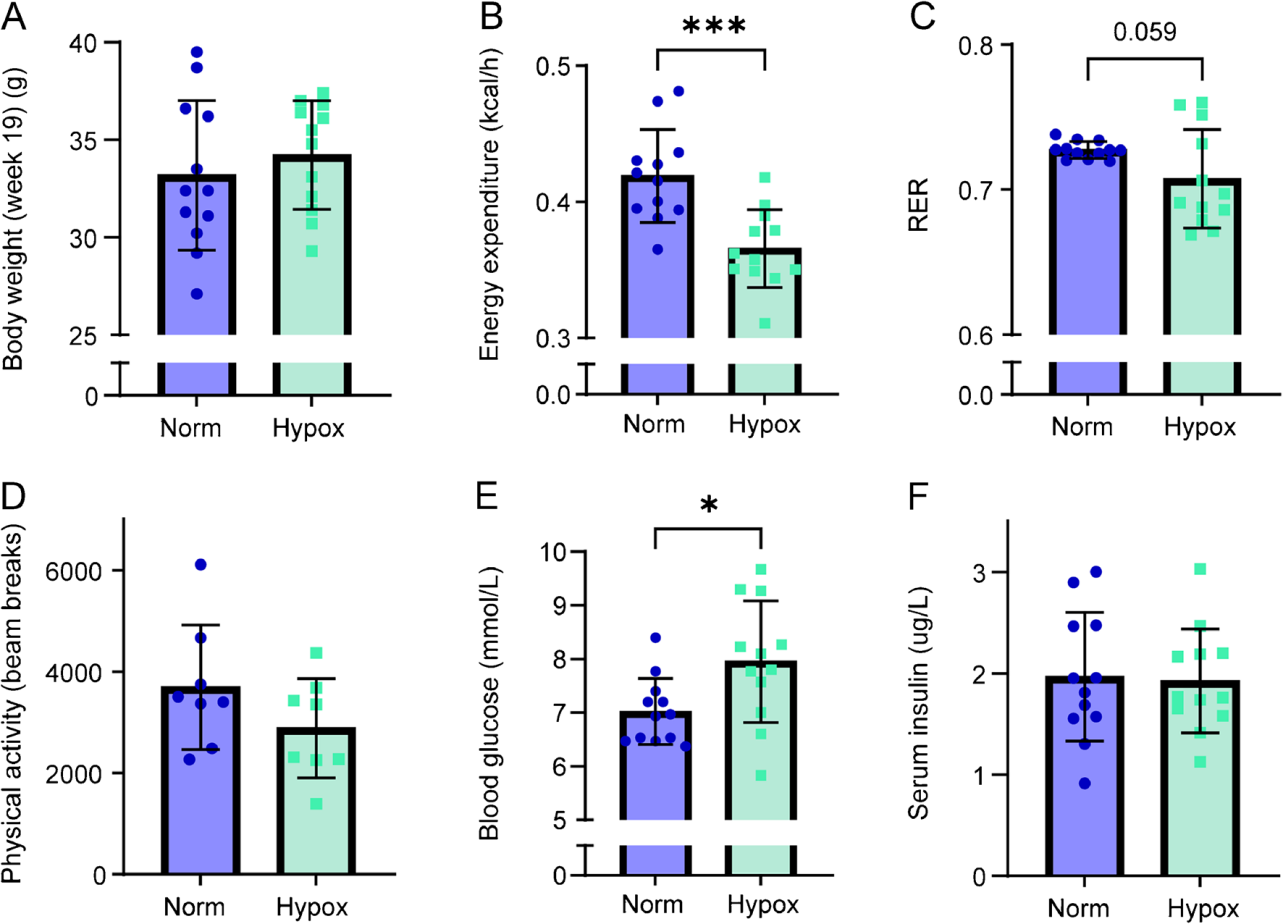
Whole body effects of six hours exposure to normoxia (Norm) and mild hypoxia (Hypox; 12% O_2_). **(a)** Body weight of the mice before exposure, **(b)** average levels of body energy expenditure and **(c)** RER during Norm or Hypox, **(d)** physical activity determined as total beam breaks, **(e)** blood glucose levels and **(f)** serum insulin levels directly after Norm or Hypox, *n* = 12 per group, *n* = 8 for physical activity, * *p*-value < 0.05, *** *p*-value < 0.001

Prior to the hypoxia (12% O_2_) exposure, mice with similar body weight (Fig. 1a, Table S2) and body composition were the assigned to the Hypox group and the Norm group (Norm mice: total fat mass 9.35 ± 2.86 g, adiposity 27.6% ± 5.7%, total lean mass 22.34 ± 1.25 g; Hypox mice: total fat mass 10.40 ± 1.88 g, adiposity 30.3% ± 4.7%, total lean mass 22.42 ± 0.84 g, Table S2). During the six hours hypoxic exposure, the average oxygen consumption (Fig. S1) and energy expenditure (Fig. 1b) were significantly lower in the Hypox group. RER, already being low due to the fasted condition, tended to be further decreased in the Hypox group (*P*-value = 0.0585) (Fig. 1c). Physical activity was similar in both groups (Fig. 1d). Due to the tendency towards a decreased physical activity, we correlated energy expenditure and RER with physical activity and found no correlation (Fig. S2), indicating that the lower energy expenditure and RER is not caused by a lower physical activity of the mice. Blood glucose levels (Fig. 1e) were significantly increased in the Hypox group, but not the serum insulin levels (Fig. 1f).

### Effects of six hours of hypoxia on skeletal muscle whole genome gene expression

**Fig. 2:**
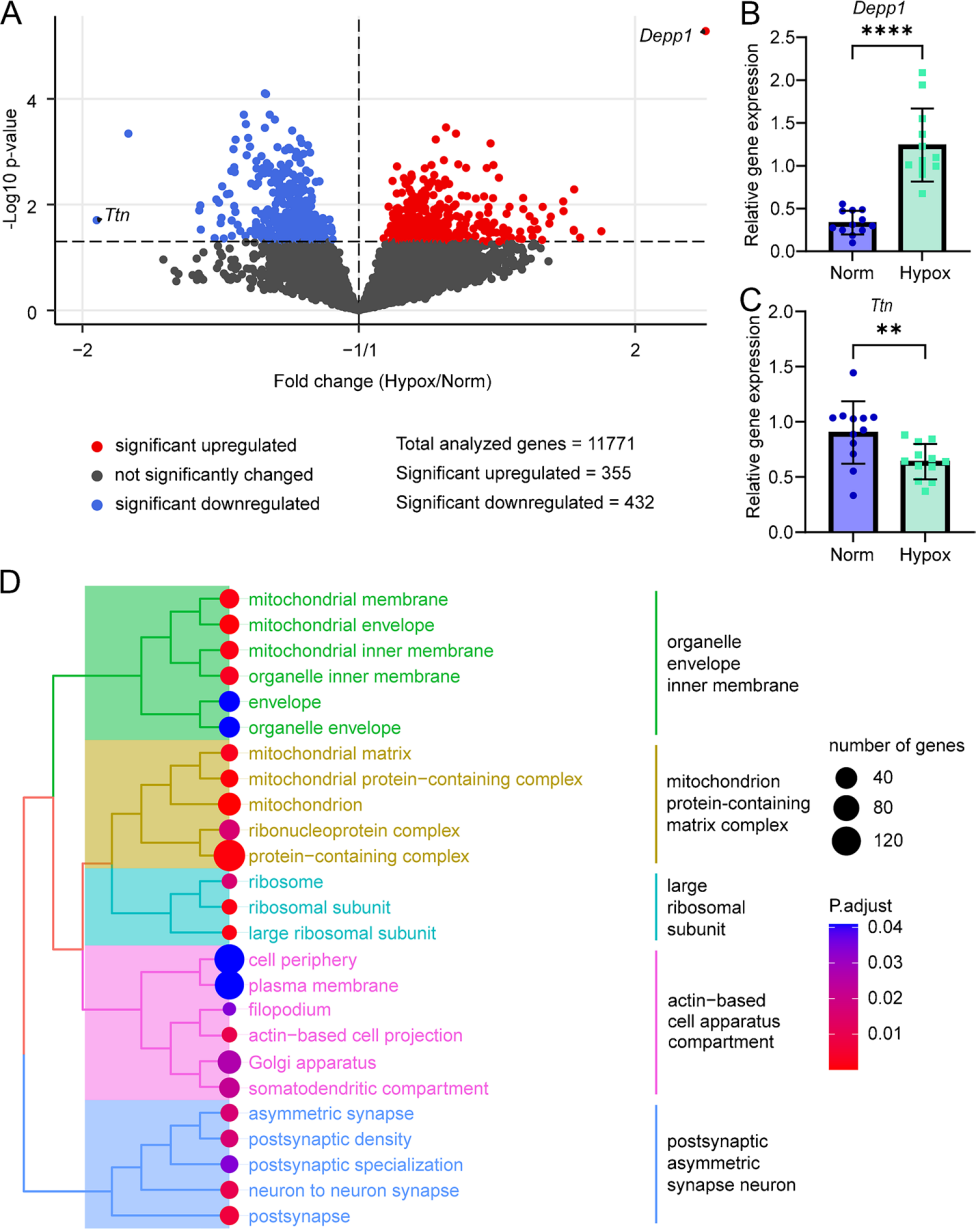
Volcano plot (**a**), confirmation (**b**, **c**) and cluster analysis (**d**) of differential expressed genes in *M. gastrocnemius* after Hypox (12% O_2_) versus Norm. **(a)** All expressed genes with an Entrez annotation are displayed in a volcano plot based on their FC and t-test *P*-value. Dashed lines indicate *P*-value < 0.05 cutoff and FC = -1/1. Non-regulated, upregulated, and downregulated genes are labelled in grey, red, and blue respectively. Q-PCR analysis of **(b)** *Depp1* and **(c)** *Ttn*. Values are represented as mean ± SD, *n* = 12 per group, ** *P*-value < 0.01, **** *P*-value < 0.0001. **(d)** Cluster analysis using gene ontology gene set enrichment using the cellular component GO-aspect (adjusted *P*-value < 0.05)

The transcriptome analysis of *M. gastrocnemius* was investigated to gain molecular insight in the consequences of acute hypoxia exposure for skeletal muscle. In total, 11,771 genes with unique Entrez annotation were detected, of which 335 genes were significantly upregulated and 432 genes were significantly downregulated in the Hypox group (Fig. 2a). *Depp1* was the most upregulated transcript (fold change in Hypox/Norm (FC) = 2.17; Fig. 2a) while *Ttn* was the most downregulated transcript (FC = -1.95; Fig. 2a). *Depp1* upregulation and *Ttn* downregulation were confirmed with Q-PCR (Fig. 2b and c; Table S3).

The gene cluster analysis, using all three aspects of GO GSEA (biological process, cellular component and molecular function), classified all significantly regulated genes into seven clusters (Fig. S3). Four of the seven clusters were based solely on the cellular component. Therefore, we performed GSEA solely with the cellular component, which resulted in five clusters (Fig. 2d). The first two clusters mainly includes terms concerning mitochondria that are organelle envelope inner membrane (36 regulated genes) and mitochondrion protein-containing matrix complex (190 regulated genes). The other three clusters are large ribosomal subunit (11 regulated genes), actin-based cell apparatus compartment (173 regulated genes), and postsynaptic asymmetric synapse neuron (25 regulated genes). These results suggest that six hours hypoxia mainly affects skeletal muscle mitochondria, skeletal muscle mitochondrial metabolism, and skeletal muscle structure, including the postsynaptic NMJ structure.

### Metabolic mitochondrial genes were significantly regulated after six hours of hypoxia

**Fig. 3:**
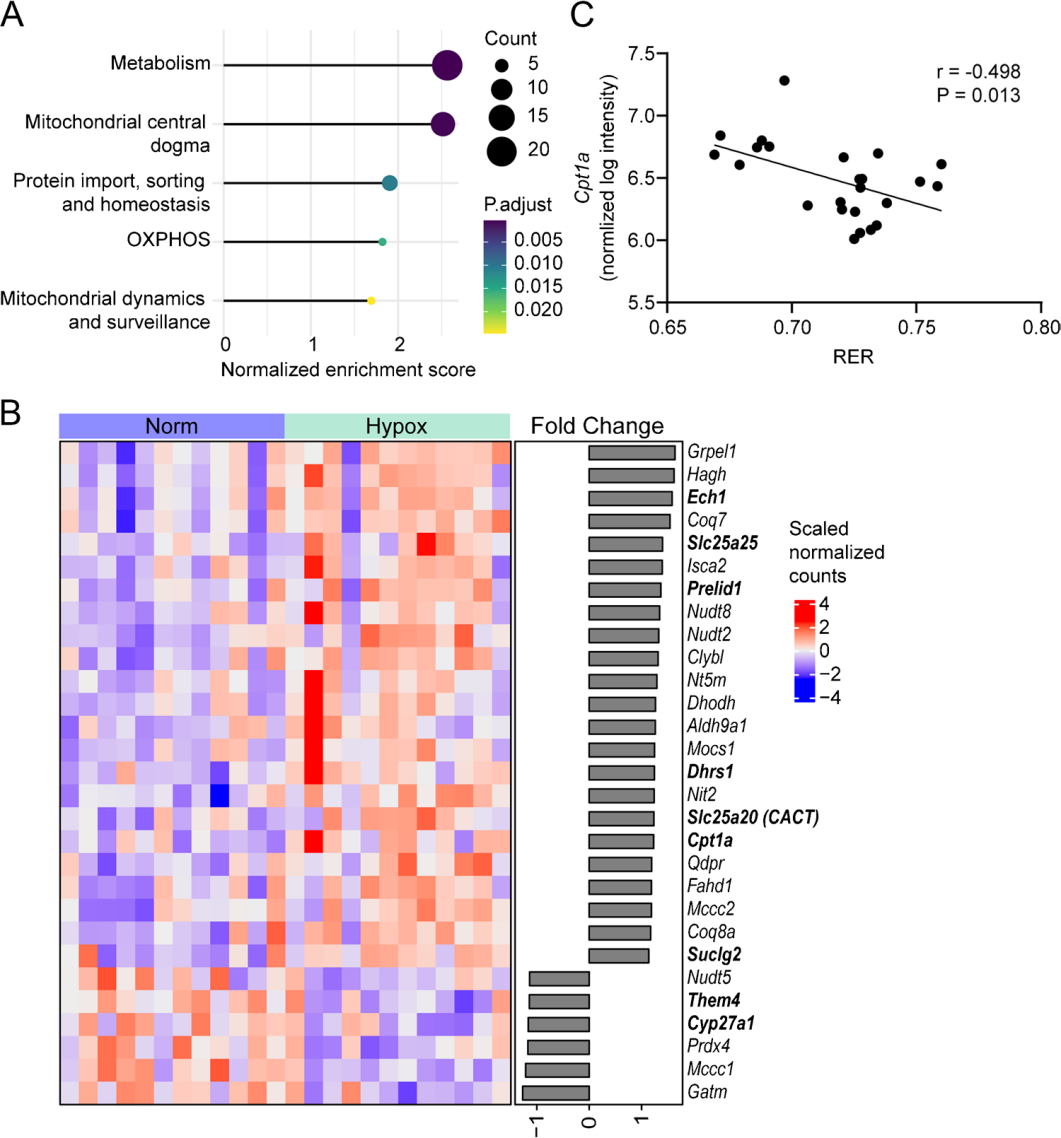
Analysis of mitochondrial genes affected by six hours Hypox (12% O_2_) versus Norm, **(a)** Gene set enrichment analysis of Hypox versus Norm using the MitoCarta 3.0 gene sets with adjusted *P*-value < 0.05 and **(b)** heatmap showing all significantly regulated genes under six hours hypoxia involved in mitochondrial metabolism using the MitoCarta 3.0 gene set, genes specifically involved in lipid metabolism are marked in bold. **(c)** Correlation between RER and *Cpt1a* expression (*n* = 24, combined Hypox and Norm)

Based on the outcome of the cluster analysis, we focused on significantly regulated mitochondrial genes, using the MitoCarta 3.0 gene list. From a total of 1,184 Mitocarta genes, 899 genes were found to be expressed. Fifty-nine of these genes were significantly regulated, of which 49 genes were significantly upregulated, and 10 genes were downregulated (Table S4). Using a normalized enrichment score, MitoCarta GSEA showed a significant upregulation of 5 out of 7 mitochondrial pathways (Fig. 3a), which were: metabolism (29 regulated genes), mitochondrial central dogma (13 regulated genes), protein import, sorting and homeostasis (6 regulated genes), OXPHOS (5 regulated genes), and mitochondrial dynamics and surveillance (6 regulated genes). MitoCarta GSEA confirmed the GO GSEA result that mitochondrial genes were regulated by hypoxia (Figs. 2d and 3a).

To obtain a better insight in the most prominently regulated mitochondrial pathway (metabolism), we sorted all regulated genes of this pathway based on their FC in descending order (Fig. 3b). Nine out of 29 genes, bold in Fig. 3b, were involved in lipid metabolism. Carnitine palmitoyltransferase 1 (CPT1) together with carnitine-acylcarnitine translocase (CACT) and CPT2 compose the carnitine shuttle. *Cpt1a* (FC = 1.23), encoding an isoform of CPT1, and *Slc25a20* (FC = 1.23), encoding CACT were upregulated in Hypox. In contrast, *Cpt1b*, the main CPT1 isoform expressed in muscle, and *Cpt2* were not changed. Correlation analysis of the normalized log intensity of muscle *Cpt1a* against whole body RER showed a negative correlation (Pearson r = -0.498, *P*-value = 0.013; Fig. 3C). Likewise, a negative correlation was also found between muscle *Cpt1a* expression and whole body energy expenditure (Pearson r = -0.495, *P*-value = 0.014; Fig. S4). To further investigate whether lipid catabolism is affected during hypoxia, acylcarnitines in the *M. gastrocnemius* were analyzed.

### Increased C12-C16 acylcarnitines after six hours of hypoxia

Table 1 shows the quantification of *M. gastrocnemius* free carnitine and 39 types of acylcarnitine. Generally, hypoxia tended to increase the amount of total acylcarnitines (FC = 1.88, *P*-value = 0.066). Especially, most C14 (C14-0, C14-1, C14-2, C14-1OH, C14-2OH), C16 (C16-0, C16-1, C16-2 and C16-1OH) and C18-1 acylcarnitines were significantly upregulated. Moreover, C18-2, C18-3, C18-1OH and C18-2OH all showed an increasing trend (*P*-value < 0.1). Additionally, concentrations of C20 acylcarnitine showed a striking increase (FC > 1.4), but without statistical significance (*P*-value > 0.1), between Hypox and Norm. Overall, total long-chain (C14-C20) acylcarnitines tended to be increased in the Hypox group (FC = 1.90; *P*-value = 0.065). Moreover, C14-1, C16-0 and C18-1 are clinical markers for an impaired beta oxidation, and these were individually and jointly, significantly upregulated. This accumulation of acylcarnitines in muscle is in line with upregulated *Cpt1a* without concomitant upregulation of downstream β-oxidation genes. Exposure to hypoxia did not affect free carnitine, neither most short-chain (C2-C5) and medium-chain (C6-C12) acylcarnitine levels, except for C5-1 carnitine (downregulated), and C5DC and C12-1 carnitine (upregulated). Altogether, the changes in acylcarnitine levels, and in particular the alterations in C16, can be considered a functional confirmation of our gene expression data.

**Table 1:** Overview of acylcarnitines and free carnitine levels in skeletal muscle after six hours Hypox (12% O_2_) versus Norm

Metabolites	Norm (nmol/g)	Hypox (nmol/g)	FC	P-value	95% confidence interval
Free carnitine					
L-carnitine	97.67 ± 25.25	92.78 ± 18.27	−1.05	0.593	−23.65, 13.88
Short-chain acylcarnitines					
C2-0	44.04 ± 5.15	44.44 ± 5.85	1.01	0.863	−4.28, 5.06
C3-0	0.55 ± 1.23	0.54 ± 1.43	−1.03	0.795	−0.14, 0.14
C4-0	2.78 ± 0.99	2.68 ± 0.62	−1.04	0.750	−0.82, 0.60
C4OH	1.99 ± 0.76	2.30 ± 0.57	1.16	0.264	−0.26, 0.89
C5-0	0.18 ± 0.05	0.15 ± 0.05	−1.21	0.128	−0.07, 0.10
C5-1	0.02 ± 1.36	0.01 ± 1.65	−1.83	**0.002**	−0.01, 0.00
C2-C5	49.58 ± 4.81	50.15 ± 5.82	1.01	0.798	−3.96, 5.10
Medium-chain acylcarnitines					
C4DC	3.09 ± 1.19	2.76 ± 1.11	−1.12	0.076	−0.64, 0.04
C6-0	0.74 ± 0.31	0.76 ± 0.25	1.02	0.884	−0.22, 0.05
C6-1	0.03 ± 1.46	0.03 ± 1.5	1.07	0.659	−0.01, 0.01
C5DC	0.44 ± 0.09	0.61 ± 0.15	1.37	**0.004**	0.06, 0.27
C8-0	0.37 ± 0.15	0.38 ± 0.13	1.05	0.756	−0.10, 0.13
C8-1	0.15 ± 0.05	0.15 ± 0.05	−1.02	0.859	−0.05, 0.04
C7DC	0.09 ± 0.04	0.13 ± 0.05	1.37	0.072	−0.00, 0.07
C10-0	0.32 ± 0.15	0.41 ± 0.15	1.28	0.161	−0.04, 0.22
C10-1	0.15 ± 0.07	0.18 ± 0.07	1.18	0.355	−0.03, 0.09
C10-2	0.04 ± 0.02	0.03 ± 0.01	−1.08	0.674	−0.01, 0.01
C12-0	0.96 ± 0.35	1.34 ± 0.57	1.40	0.064	−0.02, 0.78
C12-1	0.21 ± 0.10	0.31 ± 0.13	1.49	**0.048**	0.00, 0.20
C6-C12	6.64 ± 1.49	7.11 ± 1.42	1.07	0.434	−0.76, 1.70
Long-chain acylcarnitines					
C12DC	0.06 ± 0.03	0.09 ± 0.04	1.60	**0.031**	0.00, 0.06
C14-0	2.46 ± 1.10	3.79 ± 1.73	1.54	**0.037**	0.09, 2.57
C14-1	1.19 ± 0.47	1.89 ± 0.71	1.59	**0.010**	0.19, 1.22
C14-2	0.46 ± 0.21	0.74 ± 0.30	1.61	**0.016**	0.06, 0.50
C14OH	0.2 ± 1.77	0.32 ± 1.78	1.62	0.051	0.00, 0.33
C14-1OH	0.16 (0.13, 0.17)	0.52 (0.27, 0.55)	2.41	**0.014**	0.10, 0.40
C14-2OH	0.14 (0.09, 0.18)	0.46 (0.27, 0.54)	2.36	**0.012**	0.09, 0.41
C16-0	10.21 ± 5.88	18.26 ± 8.70	1.79	**0.015**	1.72, 14.40
C16-1	3.62 ± 1.93	6.89 ± 3.47	1.90	**0.011**	0.86, 5.69
C16-2	2.07 ± 1.17	3.52 ± 1.77	1.70	**0.030**	0.16, 2.73
C16OH	0.71 ± 1.95	1.06 ± 2.01	1.50	0.161	−0.11, 1.18
C16-1OH	0.38 (0.25, 0.46)	1.03 (0.67, 1.23)	2.12	**0.014**	0.21, 0.85
C16-2OH	0.15 ± 1.77	0.22 ± 1.79	1.47	0.117	−0.01, 0.21
C16DC	2.22 ± 1.86	2.33 ± 1.99	1.05	0.856	−0.88, 1.84
C18-0	276.6 ± 2.2	451.4 ± 2.23	1.63	0.146	−46.49, 608.62
C18-1	885.7 ± 2.23	1755 ± 2.24	1.98	**0.049**	3.90, 2576.77
C18-2	631.3 ± 2.4	1313 ± 2.37	2.08	0.051	−1.53, 2107.98
C18-3	66.35 ± 2.12	122.5 ± 2.27	1.85	0.069	−3.43, 172.18
C18OH	34.78 ± 2.28	44.61 ± 2.22	1.28	0.460	−12.31, 53.74
C18-1OH	101.6 ± 2.26	199.1 ± 2.24	1.96	0.054	−1.37, 293.85
C18-2OH	90.06 ± 2.3	161.8 ± 2.2	1.80	0.091	−8.61, 231.26
C20-0	24.29 ± 2.5	37.58 ± 2.6	1.55	0.265	−7.26, 58.60
C20-1	141 ± 2.54	250.5 ± 2.59	1.78	0.149	−28.07, 414.54
C20-2	182 ± 2.42	314.7 ± 2.5	1.73	0.150	−35.07, 492.02
C20-3	35.56 ± 2.13	53.43 ± 2.28	1.50	0.221	−8.22, 68.90
C20-4	19.02 ± 1.91	26.85 ± 2.06	1.41	0.231	−3.99, 28.94
C14-C20	2547 ± 2.26	4852 ± 2.24	1.90	0.065	−106.44, 7096.37
Total acylcarnitines	2621 ± 2.22	4924 ± 2.22	1.88	0.066	−113.23, 7045.45

Data were shown as mean ± SD, geometric mean ± geometric SD for lognormal distribution data, or median (IQR). FC representing in Hypox/Norm (*n* = 12 per group), *P*-value < 0.05 in Welch t-test or Mann–Whitney tests are marked in bold. 95% confidence interval was calculated for the difference in means (Hypox – Norm)

### FOXO signaling pathway was activated by six hours of hypoxia

**Fig. 4:**
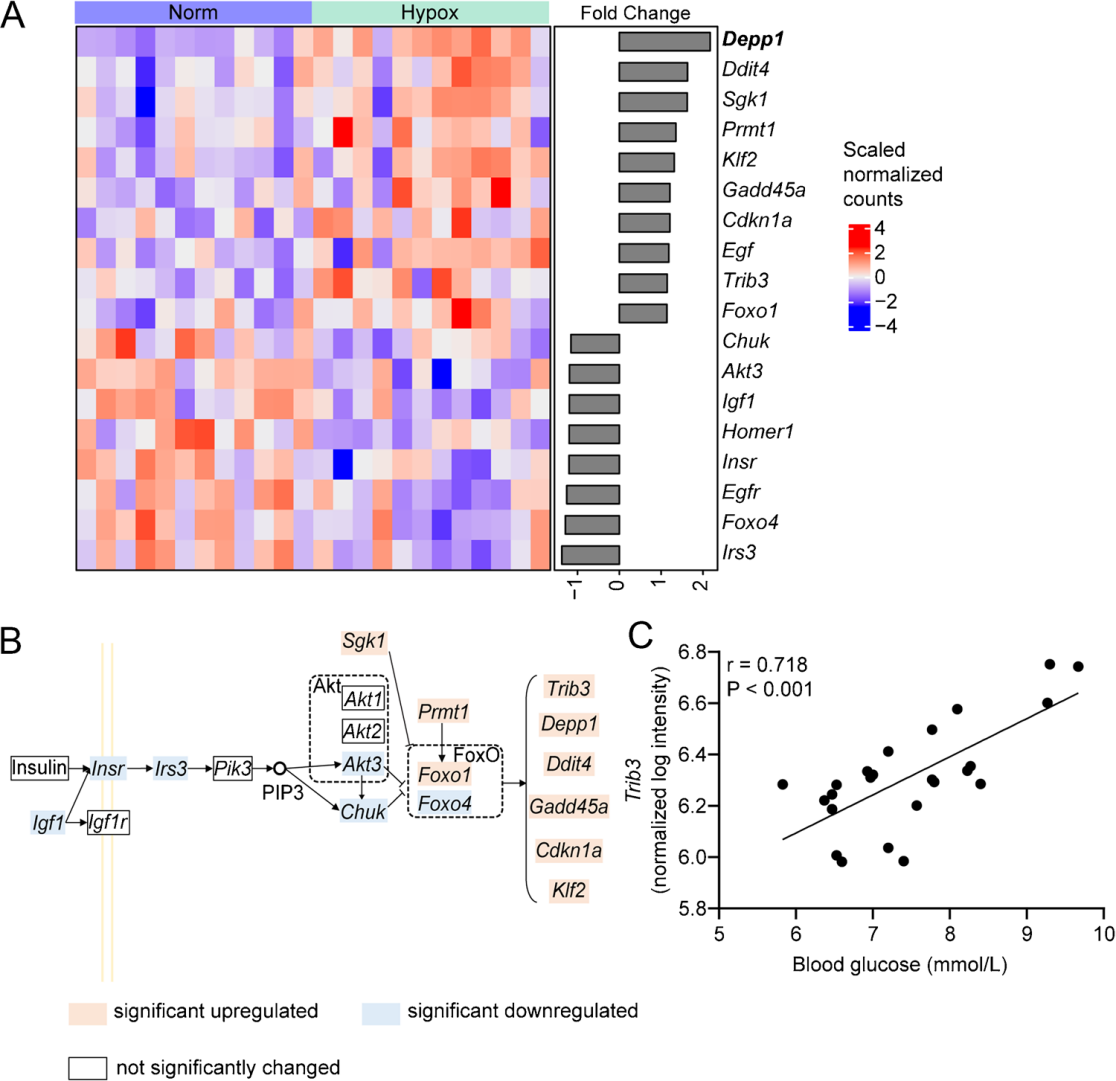
The effect of six hours hypoxia on the FOXO signaling pathway. **(a)** Heatmap showing all significantly regulated genes involved in the FOXO pathway in Hypox (12% O_2_) versus Norm. **(b)** Schematic presentation of the FOXO pathway. **(c)** Correlation between blood glucose and *Trib3* expression (*n* = 24, combined Hypox and Norm)

KEGG pathway enrichment analysis revealed that the FOXO signaling pathway was the only one being significantly regulated (adjusted *P*-value = 0.022) with a total of 15 regulated genes that are shown in a heatmap (Fig. 4a). FOXOs have a role in proteolysis and skeletal muscle atrophy [100], which is compensated by the upregulation of ribosomal genes as identified with GO GSEA (Fig. 2d). The hypoxia exposure regulated *Foxo1* and *Foxo4* gene expression in opposite directions, with an upregulation of *Foxo1* and downregulation of *Foxo4*. The integrated pathway map of the FOXO signaling pathway including regulated and non-regulated genes upstream of FOXO is shown in Fig. 4b. The function of *Foxo1* and *Foxo4* is inhibited by serum/glucocorticoid regulated kinase 1 (*Sgk1*), thymoma viral proto-oncogene 3 (*Akt3*) and conserved helix-loop-helix ubiquitous kinase (*Chuk*), of which the gene expression of *Akt3* (FC = -1.19) and *Chuk* (FC = -1.16) were downregulated and *Sgk1* (FC = 1.62) was upregulated. Additionally, insulin-like growth factor 1 (*Igf1*; FC = -1.19), insulin receptor (*Insr*; FC = -1.20) and insulin receptor substrate 3 (*Irs3*; FC = -1.20) which are involved in the activation of *Akt3* and *Chuk* were also downregulated. In addition, protein arginine N-methyltransferase 1 (*Prmt1*), stimulating FOXO, was upregulated (FC = 1.35). These results suggest that FOXO and particularly *Foxo1*, was upregulated. Since the FOXO pathway in the KEGG database is based on literature until 2013 and only included growth arrest and DNA-damage-inducible 45α (*Gadd45a*), cyclin-dependent kinase inhibitor 1A (*Cdkn1a*) and Kruppel-like factor 2 (*Klf2*), we also included the established FOXO targets: DNA-damage-inducible transcript 4 (*Ddit4*), *Depp1* and *Trib3* [77] (Fig. 4a) to investigate the downstream genes. Remarkably, the expression of all these genes were significantly increased, of which *Depp1* is the most upregulated gene in the volcano plot (Fig. 2a). In addition, *Trib3* expression was strongly correlated to blood glucose levels (Pearson r = 0.718, *P*-value < 0.001; Fig. 4c). Target genes of HIF1 were also analyzed based on the study of Benita et al*.* [10]. Results showed that of the 159 reported HIF1 genes, only 10 genes were regulated (Fig. S5). Among these 10 regulated genes, *Ddit4* and *Cdkn1a* are also FOXO target genes. Together, these results suggest an activation of the FOXO signaling pathway after six hours hypoxia exposure.

### Six hours of hypoxia tended to cause denervation of NMJ and affect contractile fiber on gene expression

**Fig. 5:**
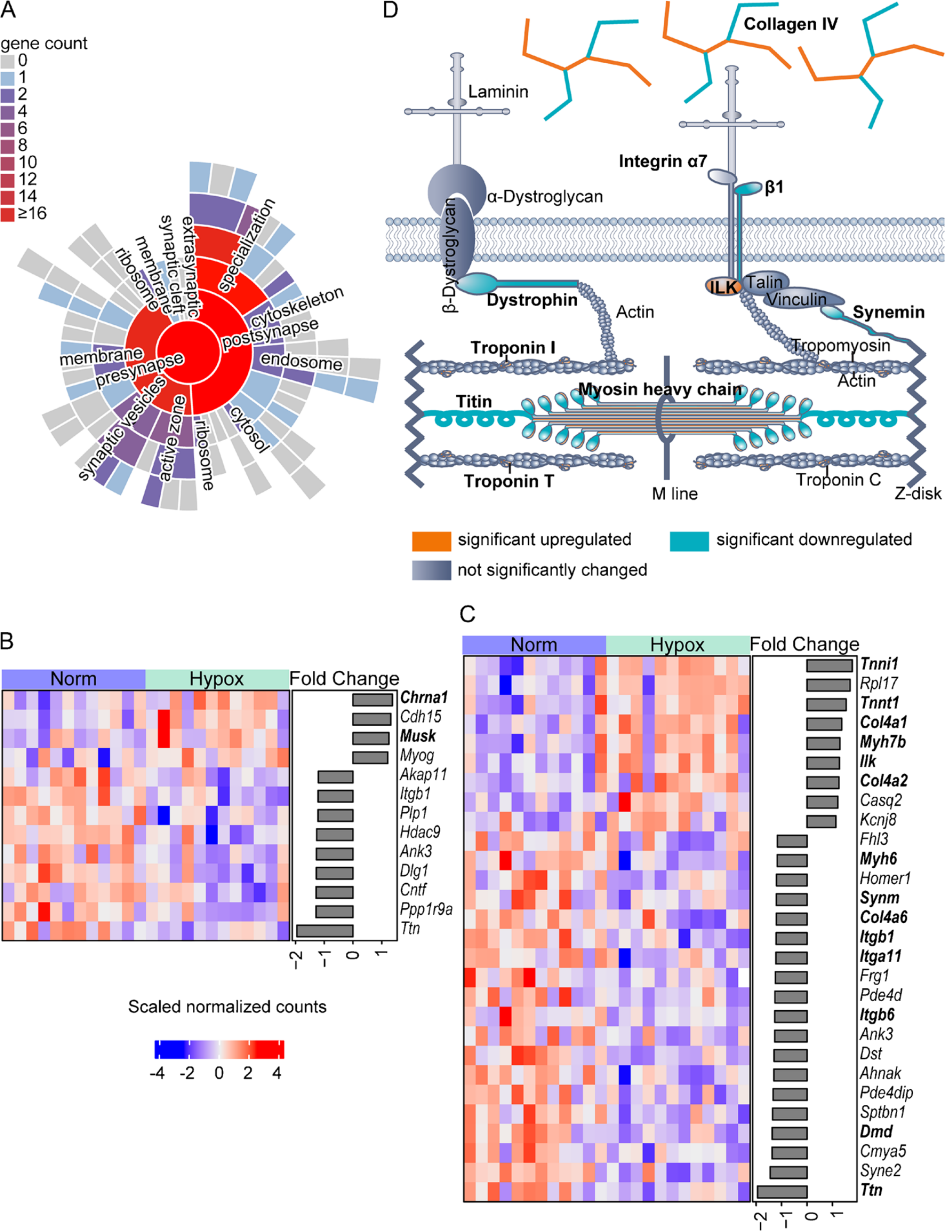
The effect of six hour Hypox versus Norm on the muscle structure, ECM and NMJ. **(a)** SynGO sunburst of the genes involved in synapse based on the cellular components including child terms **(b)** Heatmap showing significantly regulated NMJ genes after hypoxia. **(c)** Heatmap showing significantly regulated genes under hypoxia based on the GO term contractile fiber and collagen genes. **(d)** Schematic presentation of regulated genes (bold in c) that are involved in the ECM and contractile fiber

Based on the cluster of ‘postsynaptic asymmetric synapse neuron’ in the GO GSEA (Fig. 2d), we investigated the effect of six hours hypoxia exposure on genes involved in the NMJ. In total, 50 significantly regulated genes were mapped to unique SynGO annotated genes. Forty-four genes could be mapped to cellular component (Fig. 5a), and 41 genes could be mapped to the biological processes (Table S5). SynGO GSEA showed that 2 terms (modulation of chemical synaptic transmission and structural constituent of postsynapse) in biological processes and 3 terms (postsynaptic specialization, postsynaptic density and postsynaptic density, intracellular component) in cellular components were significantly regulated (adjusted *P*-value < 0.05, Table S6). Additionally, based on the NMJ gene list [9, 26, 42, 43, 52, 68, 78], 4 genes were upregulated in the Hypox versus Norm: cholinergic receptor nicotinic alpha polypeptide 1 (*Chrna1*; FC = 1.37), cadherin 15 (*Cdh15*; FC = 1.31), muscle-specific receptor tyrosine kinase (*Musk*; FC = 1.25) and myogenin (*Myog*; FC = 1.21; Fig. 5b).

The most downregulated gene *Ttn* (Fig. 2c) and the identification of the clusters actin-based cell apparatus compartment (Fig. 2d) indicated that a six hours hypoxia exposure also affected expression of genes involved in muscle structure. All regulated genes of the contractile fiber GO term and the collagen genes, which are important components of the ECM are schematically shown in Fig. 5a. Key genes related to ECM and muscle contractile fiber structure are shown in an heatmap (Fig. 5d), of which the highlighted genes are regulated by the hypoxia exposure. Of all genes involved in the thick filament, *Ttn* and myosin heavy polypeptide 6 (*Myh6*) were downregulated, while *Myh7b* was upregulated. However, in the thin filament, only 2 genes of troponin complex, troponin I1 (*Tnni1*) and troponin T1 (*Tnnt1*), were upregulated. Notably, an upregulation of *Tnni1*, *Tnnt1* and *Myh7b* and downregulation of *Myh6* are all features of a slow fiber type, which may suggest a muscle fiber change to type I. Additionally, integrin β1 (*Itgb1*) which forms the receptor of collagen IV, were downregulated. Some collagen IV genes were altered, being *Col4a1* and *Col4a2* upregulated and *Col4a6* downregulated. Other regulated genes involved in the ECM, based on MatrisomeDB are shown in Table S7. These results indicate that six hours of hypoxia tended to affect the ECM and contractile fibers, possibly shifting to oxidative, slow fibers, and denervate NMJ.

## Discussion

### Six hours of hypoxia impairs mitochondrial oxidative metabolism

In this study, we investigated the *in vivo* effects in the *M. gastrocnemius* upon a six hours environmental normobaric hypoxia (12% O_2_) exposure of male C57BL/6JOlaHsd adult obese mice. On whole body level, the six hours of hypoxia reduced energy expenditure, increased blood glucose and tended to further increase fat and decrease glucose oxidation as shown by a decreased tendency of RER. In *M. gastrocnemius*, C14-1, C16-0 and C18-1 acylcarnitines were significantly increased, suggesting decreased tissue lipid oxidation, and transcriptomic analysis revealed the FOXO signaling pathway as the only pathway being significantly upregulated. Interestingly, the data suggest that the FOXO signaling pathway is connected to the above described processes, but also to alterations in processes involved in skeletal muscle structure, ECM, tissue remodeling, and NMJ.

The decrease in oxygen consumption at the whole body level indicates a decrease in mitochondrial OXPHOS and subsequent metabolic rate. However, no decrease in OXPHOS gene expression was seen. This is different from long-term studies showing decreased expression levels of complex I and complex IV in human after 66 days at high altitude hypoxia exposure [60] and decreased complex I and IV activity and complex I protein levels in heart mitochondria of rats exposed to 11% oxygen for two weeks [34]. The switch from oxidative (mitochondrial) to glycolytic ATP production may underlie the observed decreased oxygen consumption, but the increase in blood glucose levels suggests that this is not the case. As the mice in the current study were exposed to acute hypoxia in a fasted state, the observed increased blood glucose could be triggered by a raised hepatic gluconeogenesis [20], an increased glycogen degradation and/or a decreased glucose uptake and use by skeletal muscle [33]. Part of this could be mediated by FOXO [7, 31].

FOXOs play a critical role in hepatic glucose homeostasis. Liver specific knockout of either FOXO1 alone or FOXO1/3/4 together led to lower blood glucose levels under both fasting and non-fasting conditions in mice [72], which also indicates FOXO may play an important role in obesity. To regulate blood glucose, FOXOs activate the hepatic gluconeogenic program transcriptionally [25, 72]. In contrast, FOXOs inhibit glycolysis, likely through suppression of glucokinase and pyruvate kinase gene expression [84, 109]. However, our results did not show a gene regulation of rate-limiting enzymes of glycolysis although FOXO signaling was increased in muscle (Fig. 4b). The significantly increased blood glucose levels without changes in serum insulin levels suggests a glucose intolerance state, as it was also seen in our previous hypoxia studies [27, 28]. This may be due to the strong upregulation of the FOXO target *Trib3*. TRIB3 is known to suppress insulin-stimulated glucose uptake in skeletal muscle [58]. Furthermore, *Trib3* expression positively correlated with blood glucose levels (Fig. 4c). Together with increased blood glucose levels, unaffected insulin levels (Fig. 1e) and decreased muscle expression levels of *Insr*, this suggests that a six hours hypoxia exposure decreased insulin signaling in muscle. This data is consistent with experiments with isotope-labeled glucose in humans after 2–10 days exposure to very high altitude and with studies showing that first 2 days exposure of very high altitude initially increased fasting blood glucose in humans [15, 59, 104].

The tendency of a decreased whole body RER correlated with the increased expression of *Cpt1a*, suggesting a compensatory response to elevate lipid catabolism. In addition to *Cpt1a*, *Slc25a20 (Cact)* and Enoyl CoA hydratase-1 (*Ech1*; FC = 1.58) were upregulated on the transcriptome level. ECH1 metabolizes unsaturated fatty acids with double bonds in odd-numbered positions along the carbon chain [66]. However, expression of other genes involved in β-oxidation, including the major muscle CPT1, *Cpt1b*, were not changed. Since six hours hypoxia accumulated C12-C16 acylcarnitines, especially the clinical diagnostic tissue C14-1, C16-0 and C18-1 acylcarnitines, in the muscle tissue, this suggests an incomplete β-oxidation. Therefore, the upregulation of *Cpt1a* and *Slc25a20* was most likely an attempt to amend the reduced ATP availability and the decreased RER might reflect a strong reduction in glucose oxidation rather than an increased lipid oxidation, together with reduced metabolic rate (energy expenditure). This is in line with other studies of hypoxia exposure that showed suppressed fatty acid catabolism in skeletal muscle [74]. These results contrasted with other long-term studies that showed the opposite results, with hypoxia enhancing fatty acid β-oxidation in mouse after 8-week high-fat-diet (HFD) at 4300 m [93] and in humans after a 16-day high-altitude hypoxia (5260 m) [19].

Upon hypoxia exposure, the most upregulated transcript in the *M. gastrocnemius* was *Depp1* (Fig. 2b). *Depp1* was previously shown to be upregulated by hypoxia in kidney and brain [94], and to be mediated by FOXO1 in endothelial cells and skeletal muscle [77, 94]. DEPP1 is a critical stimulator of autophagy, a highly conserved catabolic process, which removes damaged and redundant cell components to promote survival and to counteract nutrient and energy shortage [83]. DEPP1 was shown to upregulate autophagy via the induction of reactive oxygen species (ROS) [83]. Interestingly, mild hypoxia has been associated with increase ROS production, while ROS production is decreased with severe hypoxia in cells [85]. Although it was not evident from our data, possibly because of the short duration of hypoxic exposure, it is tempting to speculate that the observed increase in *Depp1*, likely mediated by FOXO signaling, occurred to protect skeletal muscle against energy shortage, by altering ROS-mediated signaling and concomitantly increasing autophagy.

Overall, our results show that six hours hypoxia (12% O_2_) exposure of mice significantly decreased energy expenditure, with a concomitant increase in plasma glucose. The upregulation of *Depp1* and mitochondrial pathways may possibly be a response to restore energy status (Fig. 3a). However, no major alterations in core energy metabolic gene expression were observed.

### The central role of FOXO signaling

Our transcriptome-based KEGG pathway analysis identified the FOXO signaling pathway as the only significantly regulated pathway that was affected in the *M. gastrocnemius* upon a six hours exposure of mice to environmental normobaric hypoxia (12% O_2_). This agrees with a study in skeletal muscle of fish, where the FOXO signaling pathway was significantly enriched in the hypoxia tolerant group, with *Foxo1* being upregulated [18]. In mouse skeletal muscle, Gan et al*.* showed that after 2-h hypoxic (8% O_2_) exposure, AKT was dephosphorylated, which may promote downstream activation of FOXO [32]. Likewise, we found downregulated upstream genes related to phosphorylation state of FOXO, such as *Igf1*, *Insr*, *Irs3*, *Akt3* and *Chuk*. Indeed, acute liver-specific knockout of *Insr* enhanced FOXO1 activity and glucose intolerance [96]. Next to phosphorylation, FOXO1 can be methylated by PRMT1, which promotes the nuclear retention of FOXO1 by blocking the insulin/AKT-mediated phosphorylation at adjacent serine residues [106]. Similar to our result of increased *Prmt1* expression, Bayen et al*.* found increased PRMT1 expression, which in turn increased FOXO1 nuclear translocation and caused a decrease in glucose uptake and hyperglycemia in rats exposed to hypobaric hypoxia [8]. Importantly, we found that *Trib3, Depp1, Ddit4, Gadd45a, Cdkn1a, and Klf2,* all key downstream genes of FOXO are being upregulated after 6 h hypoxia exposure. Previously, TRIB3 was shown to be induced by hypoxia (0.1–0.5% O_2_) in breast cancer cells [13] and in rat pulmonary artery smooth muscle cells (5% O_2_) [16], Expression of *Ddit4* and *Gadd45a* were previously shown to be increased under hypoxia and oxidative stress [62, 64, 88]. Though *Ddit4* and *Cdkn1a* can be regulated by HIF1, analysis of HIF1 target genes supports that not HIF1, but FOXO1 may be the main regulator of skeletal muscle gene expression after 6 h hypoxia, which agrees to the results of Gan et al*.* [32]. Together our results indicates that six hours of normobaric hypoxia resulted in increased FOXO1 signaling in skeletal muscle, of which several regulated FOXO downstream genes are connected to blood glucose regulation (*Trib3*) and lipid handling as discussed previously. Strikingly, FOXO can also regulate muscle regeneration (via *Cdkn1a* and *Klf2*), the ECM [105] and NMJ (*Chrna1*) [11], which cumulatively suggests FOXO being involved in regulating all these processes. Long-term hypoxia, on the contrary, activated AKT and thereby inhibited FOXO1 in obese mice [93, 103]. It would therefore be interesting to study the effects of longer exposure and well as earlier timepoints on FOXO signaling. Due to the 6-week 40en% fat diet to mimic human fat consumption, adiposity of these mice was 30% at the time of hypoxia exposure. Therefore, these mice may be considered obese according to the WHO definition of human obesity and the lack of a specific definition for mouse obesity [24] and our results may not represent effects of acute hypoxia exposure in lean mice.

### Hypoxia, FOXO and skeletal muscle structure

In this study, we observed regulation of several genes featured in thin and thick filaments of type I (oxidative) muscle fiber (Fig. 5c), of which the upregulation of *Tnni1*, *Tnnt1* and *Myh7b* indicated that six hours hypoxia stimulated a switch to type I muscle fiber. Our findings agreed with observations in longer-term hypoxia exposure. For example, three-day hypoxia (4% O_2_) treatment remarkably elevated gene and protein expression of oxidative type I myosin heavy chain isoform in C2C12 cells [92]. Studies also found that high-altitude native deer mice have more type I fibers in the *M. gastrocnemius* [17, 65, 86]. Mechanistically, increased *Foxo1*, *Cpt1a* or *Trib3* has shown to significantly alter type I and type II muscle fiber proportions in mice [57]. Consistent with our results, overexpression of *Trib3* increased the number of type I fibers threefold in *M. soleus* and increased expression of *Myh7* and *Myh7b* in *M. gastrocnemius* [3]. Moreover, overexpression of *Cpt1a* showed a 28% increase in succinate dehydrogenase positive fibers indicating more oxidative capacity of the fibers [35]. However, overexpression of *Foxo1* reduced the number of type I fibers and the size of type I and type II fibers in skeletal muscle [48].

Long-term hypoxia-induced changes of muscle fibers and excessive accumulation of ECM were observed in skeletal muscle [95, 98]. In addition, FOXO1/3 have been shown to ameliorate fibrosis characterized by ECM deposition in various organs, including the heart, liver, lung, and kidney [105]. In skeletal muscle, FOXO was found to be necessary for the cancer-induced downregulation of ECM genes [46]. Here we observed an increased FOXO signaling (Fig. 4b) as well as ECM alterations (Table S7). Our results strongly suggested six hour hypoxia indicated long-term hypoxia effect on ECM.

Maintenance of muscle mass and function also depends on other processes, including differentiation, regeneration and innervation and hypoxia was shown to represses differentiation of myoblasts [79]. FOXO also has a role in skeletal muscle differentiation since it is a regulator of myoblast fusion and of the skeletal muscle terminal differentiation program [1]. We observed links of FOXO with skeletal muscle regeneration since the downstream genes of FOXO signaling [67, 69] are upregulated (*Klf2* and *Cdkn1a*). KLF2 plays a central role in the activation and phenotypic determination of various immune cell types, particularly those related to skeletal muscle regeneration [23]. Under hypoxia, transcription of *Cdkn1a* that inhibits differentiation and cell cycle can be enhanced by hypoxia response elements [45]. Next to FOXO, *Cdkn1a* can also be activated by myogenin (MYOG) which is activated by hypoxia in C2C12 cells [110] and involved in skeletal muscle differentiation by promoting cell cycle exit [67, 91]. In this study, *Cdkn1a* and *Myog* were upregulated. MYOG was increased in denervated skeletal muscle, and MYOG inhibition could alleviate denervation-induced muscle atrophy [67]. Therefore, the activated downstream gene of FOXO1, *Cdkn1a,* can be linked to NMJ denervation. This NMJ link was strengthened by the observed upregulation of *Chrna1*, encoding the acetylcholine receptor subunit alpha, which has been identified as a FOXO target in muscle [11]. Notably, expression of NMJ genes *Chrna1*, *Cdh15*, *Musk* and *Myog* has also been observed in denervated skeletal muscle [21, 67, 75, 90]. Thus, our results indicates that hypoxia inhibits muscle regeneration and results in denervated NMJ via FOXO signaling.

## Conclusion

Six hours exposure to normobaric hypoxia (12% O_2_) activates FOXO signaling in *M. gastrocnemius* of adult obese mice, which may link to the observed reduced oxidative metabolism and concomitant increase in serum glucose levels without increased fatty acid catabolism in muscle. Moreover, a possible impact on autophagy exists via the most upregulated transcript *Depp1*. Importantly, the hypoxia-induced FOXO activation may also be connected to fiber type shift, ECM remodeling, muscle differentiation and the NMJ, which are all involved in skeletal muscle structure. These observations, despite being descriptive, suggests that even a six hours exposure to 12% O_2_ environmental hypoxia can initiate alterations in skeletal muscle function and remodeling with a central role for FOXO. Thus, as it is observed in sleep apnea, aging, diabetes, and bouts of hypoxia during moderate activity, hypoxia may contribute to tissue remodeling, ultimately contributing to a lower quality of life. In particular, since intact NMJ are a key to muscle activation and essential to prevent muscle wasting, the observed impact on innervation of the NMJ is a novel result that deserves further investigation.

### Supplementary Information

Below is the link to the electronic supplementary material.

Supplementary file1 (DOCX 632 KB)

## Data Availability

All arrays were deposited in Gene Expression Omnibus (GEO) with accession ID: GSE228719.
