# Supplementary material for: Normobaric hypoxia shows enhanced FOXO1 signaling in obese mouse gastrocnemius muscle linked to metabolism and muscle structure and neuromuscular innervation
Source: Pflugers Arch. 2023 Sep 1;475(11):1265–81. doi: 10.1007/s00424-023-02854-4 (PMC10567817; doi:10.1007/s00424-023-02854-4)
Supplement: Supplementary file 1 — Supplementary file1 (DOCX 632 KB) [file 424_2023_2854_MOESM1_ESM.docx]

Supplementary data

**Table S1:** Primers for Q-PCR

| Symbol | RefSeq | Forward Primer (F, 5’-3’)  Reverse Primer (R, 5’-3’) | Product length |
| --- | --- | --- | --- |
| *Depp1* | NM_145980.2 | F: TAGCTCCCACAATGCGACTT  R: GGTGGTCACTCCACCTACAC | 127 |
| *Ttn* | NM_011652.3 | F: CCAAAGGAAGAGGTGGTGCT  R: GAGGTGGCTGTGGCTCTTC | 118 |
| *B2M*  *(ref)* | NM_009735 | F: CCCCACTGAGACTGATACATACGC  R: AGAAACTGGATTTGTAATTAAGCAGGTTC | 148 |
| *Canx*  *(ref)* | NM_007597 | F: GCAGCGACCTATGATTGACAACC  R: GCTCCAAACCAATAGCACTGAAAGG | 170 |
| *Rps15*  *(ref)* | NM_009091 | F: CGGAGATGGTGGGTAGCATGG  R: ACGGGTTTGTAGGTGATGGAGAAC | 118 |

**Table S2:** P-value and 95% confidence interval of the difference between Hypox and Norm

|  | Norm | Hypox | P-value | 95% confidence interval |
| --- | --- | --- | --- | --- |
| Body weight (g) | 33.18 ± 3.84 | 34.22 ± 2.78 | 0.459 | -1.82, 3.89 |
| Total fat mass (g) | 9.35 ± 2.86 | 10.40 ± 1.88 | 0.301 | -1.02, 3.12 |
| Adiposity % | 27.6 ± 5.7 | 30.3 ± 4.7 | 0.216 | -1.70, 7.10 |
| Total lean mass (g) | 22.34 ± 1.25 | 22.42 ± 0.84 | 0.856 | -0.83, 0.99 |
| Oxygen consumption (ml/h) | 88.08 ± 6.98 | 76.84 ± 6.10 | **0.000** | -16.79, -5.70 |
| Energy expenditure (kcal/h) | 0.42 ± 0.03 | 0.37 ± 0.03 | **0.000** | -0.08, -0.03 |
| RER | 0.73 ± 0.01 | 0.71 ± 0.03 | 0.059 | -0.04, 0.00 |
| Physical activity (beam breaks) | 3694 ± 1227 | 2882 ± 977.6 | 0.165 | -2001, 377.7 |
| Blood glucose (mmol/L) | 7.02 ± 0.62 | 7.95 ± 1.13 | **0.023** | 0.14, 1.71 |
| Serum insulin (ug/L) | 1.97 ± 0.63 | 1.93 ± 0.51 | 0.867 | -0.53, 0.45 |

Data were shown as mean ± SD. 95% confidence interval was calculated for the difference in means (Hypox – Norm).


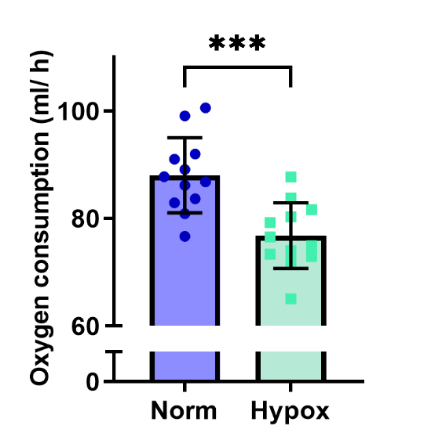


**Fig. S1:** Average levels of oxygen consumption during the six hours of Norm or Hypox (n=12 per group) *** P-value < 0.001


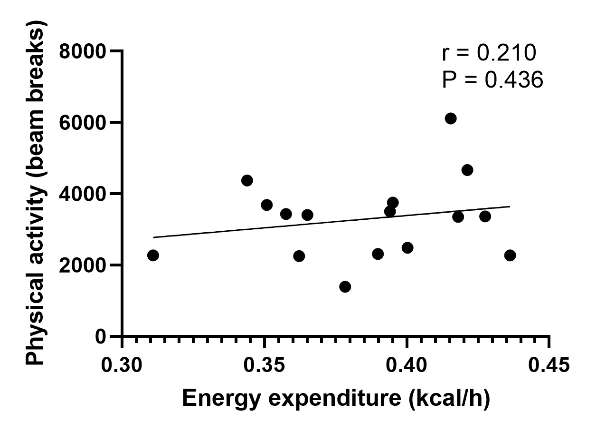

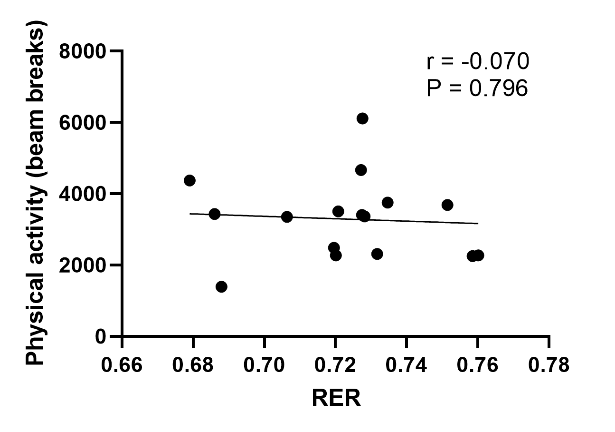


A

B

**Fig. S2:** Correlations of energy expenditure **(a)** and RER **(b)** with physical activity (n=16, combined Hypox and Norm)

**Table S3:** P-value and 95% confidence interval of gene expression of Q-PCR analysis

| Relative gene expression | Norm | Hypox | P-value | 95% confidence interval |
| --- | --- | --- | --- | --- |
| *Depp1* | 0.34 ± 0.14 | 1.24 ± 0.43 | **<0.000** | 0.64, 1.17 |
| *Ttn* | 0.90 ± 0.28 | 0.64 ± 0.16 | **0.010** | -0.46, 0.07 |

Data were shown as mean ± SD. 95% confidence interval was calculated for the difference in means (Hypox – Norm).


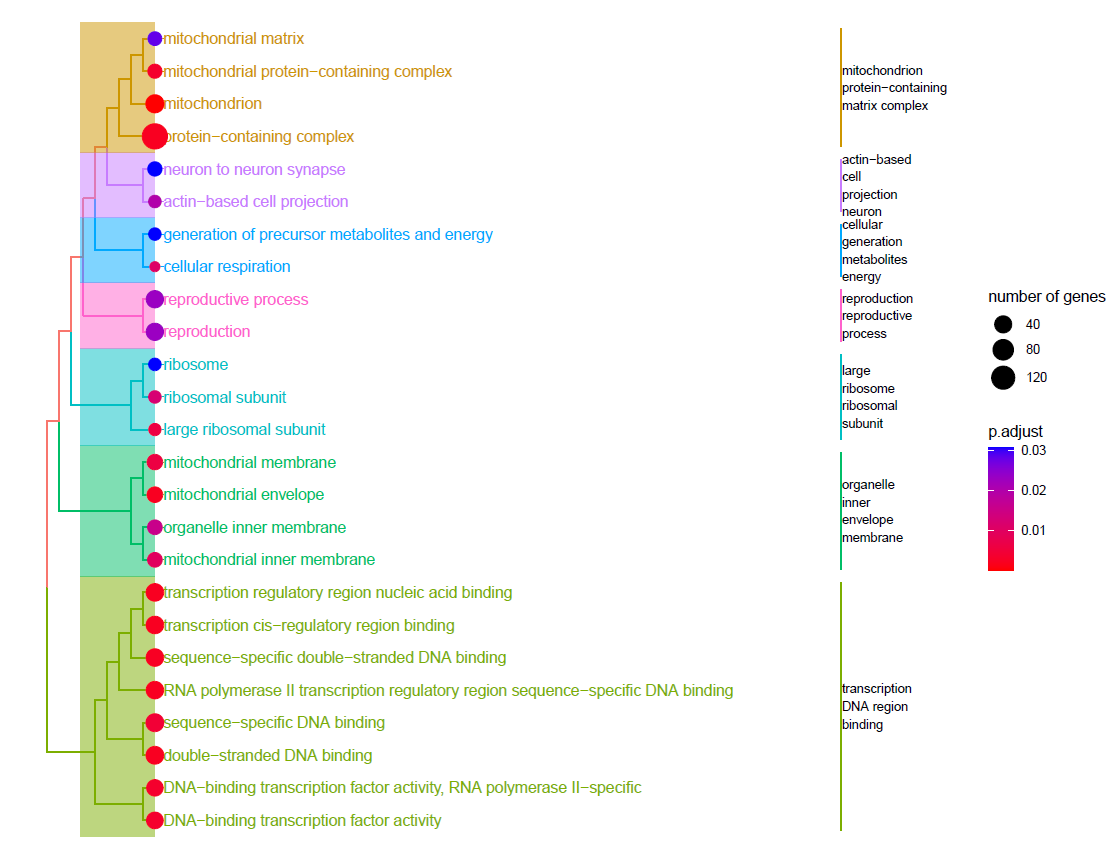


**Fig. S3:** The seven clusters classified from gene set enrichment analysis of six hours hypoxia versus normoxia using all 3 aspects in gene ontology gene list (biological process, cellular component and molecular function), adjusted P-value of 0.05

**Table S4:** Overview of differently regulated mitochondrial genes after six hours of hypoxia in MitoCarta 3.0

| Gene | MitoPathway | Fold change | P-value |
| --- | --- | --- | --- |
| *Grpel1* | Metabolism & Protein import, sorting and homeostasis | 1.64 | 0.021 |
| *Hagh* | Metabolism | 1.62 | 0.017 |
| *Ech1* | Metabolism | 1.58 | 0.044 |
| *Coq7* | Metabolism | 1.54 | 0.031 |
| *Slc25a25* | Metabolism & Signaling | 1.40 | 0.015 |
| *Isca2* | Metabolism | 1.39 | 0.038 |
| *Prelid1* | Metabolism | 1.37 | 0.028 |
| *Nudt8* | Metabolism | 1.34 | 0.040 |
| *Nudt2* | Metabolism | 1.33 | 0.004 |
| *Clybl* | Metabolism | 1.31 | 0.011 |
| *Nt5m* | Metabolism | 1.29 | 0.035 |
| *Dhodh* | Metabolism | 1.26 | 0.027 |
| *Aldh9a1* | Metabolism | 1.26 | 0.042 |
| *Mocs1* | Metabolism | 1.25 | 0.019 |
| *Dhrs1* | Metabolism | 1.24 | 0.006 |
| *Nit2* | Metabolism | 1.24 | 0.005 |
| *Slc25a20* | Metabolism & Small molecule transport | 1.23 | 0.020 |
| *Cpt1a* | Metabolism | 1.23 | 0.012 |
| *Qdpr* | Metabolism | 1.19 | 0.015 |
| *Fahd1* | Metabolism | 1.19 | 0.035 |
| *Mccc2* | Metabolism | 1.19 | 0.042 |
| *Coq8a* | Metabolism | 1.17 | 0.049 |
| *Suclg2* | Metabolism | 1.14 | 0.034 |
| *Nudt5* | Metabolism | -1.14 | 0.042 |
| *Them4* | Metabolism | -1.14 | 0.019 |
| *Cyp27a1* | Metabolism | -1.16 | 0.043 |
| *Prdx4* | Metabolism | -1.17 | 0.044 |
| *Mccc1* | Metabolism | -1.21 | 0.002 |
| *Gatm* | Metabolism | -1.27 | 0.021 |
| *Trub2* | Mitochondrial central dogma | 1.67 | 0.011 |
| *Mrpl50* | Mitochondrial central dogma | 1.53 | 0.044 |
| *Endog* | Mitochondrial central dogma & Mitochondrial dynamics and surveillance | 1.50 | 0.034 |
| *Mrpl28* | Mitochondrial central dogma | 1.49 | 0.008 |
| *Mrpl46* | Mitochondrial central dogma | 1.35 | 0.016 |
| *Mrps35* | Mitochondrial central dogma | 1.27 | 0.003 |
| *Mrpl38* | Mitochondrial central dogma | 1.27 | 0.027 |
| *Mtif3* | Mitochondrial central dogma | 1.25 | 0.032 |
| *Mpv17l2* | Mitochondrial central dogma | 1.23 | 0.044 |
| *Mrpl37* | Mitochondrial central dogma | 1.21 | 0.039 |
| *Mrpl49* | Mitochondrial central dogma | 1.19 | 0.003 |
| *Mrps23* | Mitochondrial central dogma | 1.15 | 0.026 |
| *Rcc1l* | Mitochondrial central dogma | 1.14 | 0.020 |
| *Dnajc15* | Protein import, sorting and homeostasis | 1.80 | 0.042 |
| *Grpel1* | Protein import, sorting and homeostasis | 1.64 | 0.021 |
| *Samm50* | Protein import, sorting and homeostasis & Mitochondrial dynamics and surveillance | 1.37 | 0.033 |
| *Phb2* | Protein import, sorting and homeostasis | 1.36 | 0.002 |
| *Tomm40l* | Protein import, sorting and homeostasis | 1.21 | 0.033 |
| *Timm10b* | Protein import, sorting and homeostasis | 1.18 | 0.034 |
| *Ndufb6* | OXPHOS | 1.66 | 0.047 |
| *Ndufa10* | OXPHOS | 1.55 | 0.042 |
| *Ndufaf7* | OXPHOS | 1.33 | 0.046 |
| *Lyrm2* | OXPHOS | 1.27 | 0.019 |
| *Timmdc1* | OXPHOS | -1.14 | 0.028 |
| *Mcl1* | Mitochondrial dynamics and surveillance | 1.44 | 0.026 |
| *Chchd6* | Mitochondrial dynamics and surveillance | 1.18 | 0.044 |
| *Armc10* | Mitochondrial dynamics and surveillance | 1.12 | 0.030 |
| *Marchf5* | Mitochondrial dynamics and surveillance | -1.23 | 0.022 |
| *Pptc7* | Signaling | -1.14 | 0.027 |
| *Ucp3* | Small molecule transport | 1.19 | 0.024 |
| *Abca9* | Small molecule transport | -1.34 | 0.014 |

Data are expressed as the fold changes of the level in Hypox versus Norm (n=12 per group)


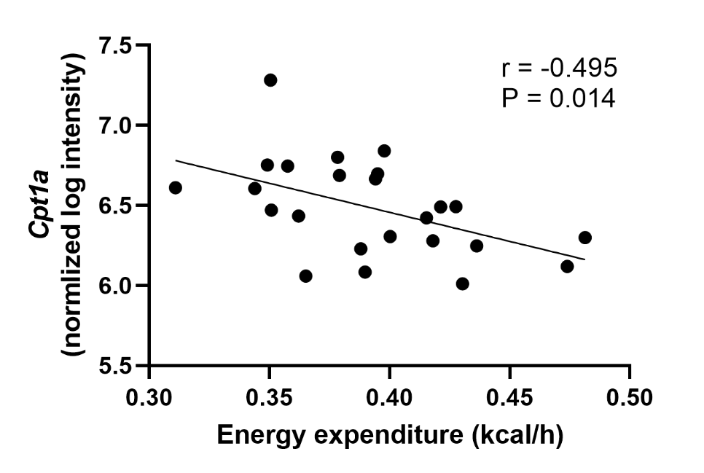


**Fig. S4:** Correlation between energy expenditure and *Cpt1a* expression (n=24, combined Hypox and Norm)


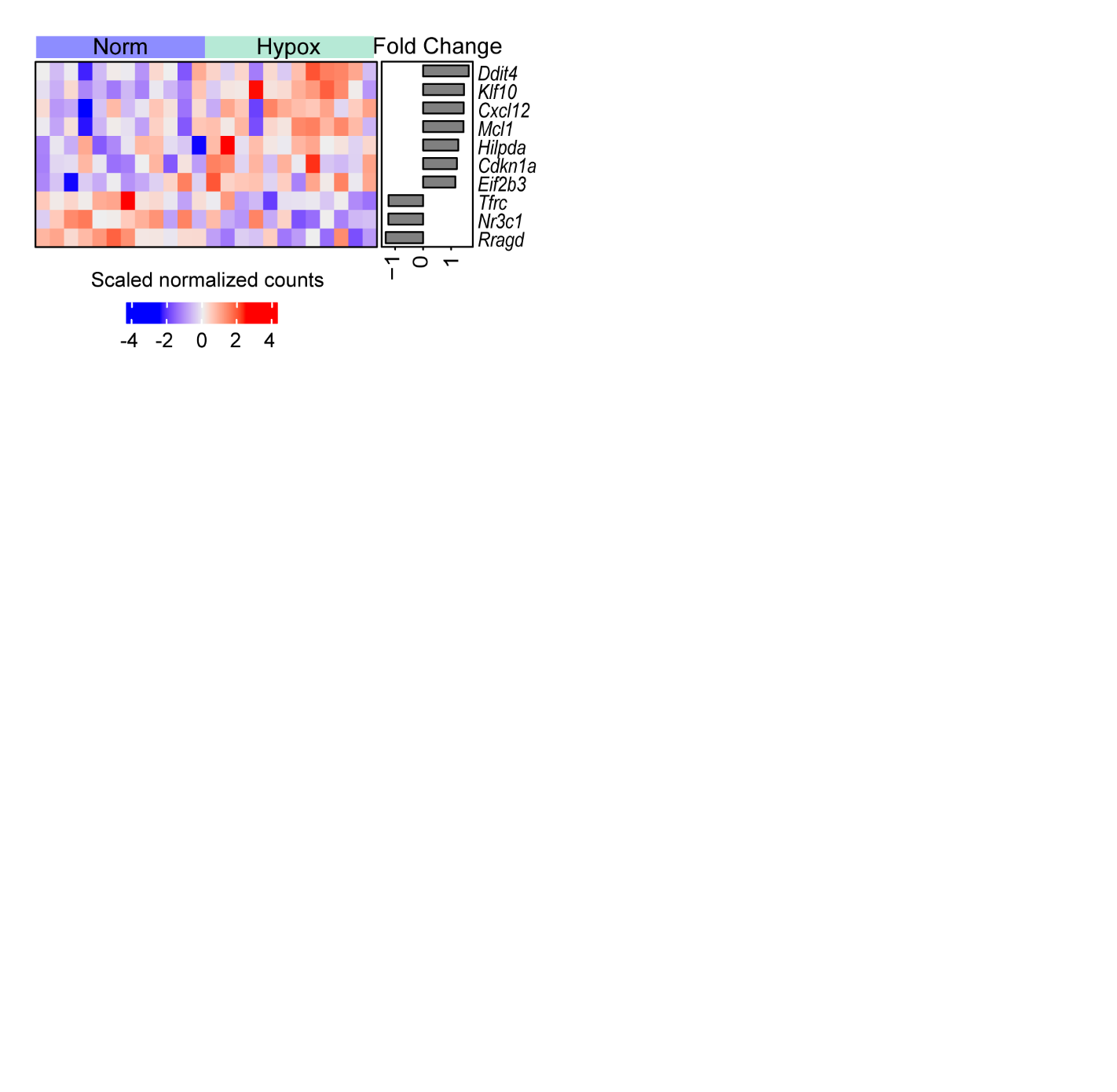


**Fig. S5:** Regulated HIF1 target genes after six hours of hypoxia

**Table S5:** Overview of differently regulated SynGO annotated genes after six hours of hypoxia

| Gene | GO aspect | GO term | Fold change | P-value |
| --- | --- | --- | --- | --- |
| *Abhd17a* | BP & CC | regulation of postsynapse organization; anchored component of postsynaptic density membrane; anchored component of postsynaptic recycling endosome membrane | 1.16 | 0.042 |
| *Abr* | BP & CC | modulation of chemical synaptic transmission; postsynaptic density, intracellular component | -1.32 | 0.048 |
| *Akap7* | BP | modulation of chemical synaptic transmission | -1.45 | 0.002 |
| *Arhgef9* | BP & CC | regulation of postsynaptic specialization assembly; postsynaptic specialization | -1.32 | 0.016 |
| *Atp2b1* | CC | integral component of presynaptic active zone membrane; presynaptic membrane | -1.16 | 0.045 |
| *Atp6v1c1* | BP & CC | synaptic vesicle proton loading; extrinsic component of synaptic vesicle membrane | 1.39 | 0.023 |
| *Bcr* | BP & CC | modulation of chemical synaptic transmission; postsynaptic density, intracellular component | -1.17 | 0.011 |
| *Camk2b* | BP & CC | regulation of synapse maturation; structural constituent of postsynaptic actin cytoskeleton; postsynaptic density | -1.26 | 0.022 |
| *Chrna1* | BP & CC | transmitter-gated ion channel activity involved in regulation of postsynaptic membrane potential; integral component of postsynaptic specialization membrane | 1.37 | 0.024 |
| *Cnih2* | BP & CC | regulation of postsynaptic neurotransmitter receptor activity; integral component of postsynaptic density membrane | -1.28 | 0.009 |
| *Cpeb1* | BP & CC | modulation of chemical synaptic transmission; regulation of translation at synapse, modulating synaptic transmission; postsynapse; postsynapse density | -1.22 | 0.047 |
| *Diaph1* | BP | synaptic vesicle endocytosis | -1.22 | 0.013 |
| *Dlg1* | BP & CC | neurotransmitter receptor localization to postsynaptic specialization membrane; structural constituent of postsynaptic density; postsynaptic density, intracellular component | -1.27 | 0.005 |
| *Dmd* | CC | postsynaptic specialization | -1.37 | 0.002 |
| *Dtnbp1* | BP & CC | regulation of synaptic vesicle cycle; regulation of synaptic vesicle exocytosis; postsynaptic density; synaptic vesicle membrane | 1.24 | 0.021 |
| *Ehd1* | BP & CC | synaptic vesicle endocytosis; presynapse | 1.49 | 0.022 |
| *Flot1* | BP & CC | regulation of neurotransmitter uptake; presynapse; presynaptic active zone; synapse | 1.41 | 0.042 |
| *Hnrnpf* | CC | synapse | -1.43 | 0.032 |
| *Homer1* | BP & CC | regulation of postsynaptic neurotransmitter receptor activity; structural constituent of postsynapse; postsynaptic cytosol; postsynaptic density | -1.20 | 0.005 |
| *Igf1* | BP & CC | postsynaptic modulation of chemical synaptic transmission; neuronal dense core vesicle lumen | -1.19 | 0.011 |
| *Igsf9b* | BP & CC | synapse adhesion between presynapse and postsynapse; postsynaptic specialization of symmetric synapse | -1.15 | 0.045 |
| *Ina* | BP & CC | postsynaptic modulation of chemical synaptic transmission; structural constituent of postsynaptic intermediate filament cytoskeleton; postsynapse; postsynaptic density, intracellular component; postsynaptic intermediate filament cytoskeleton | -1.22 | 0.032 |
| *Itgb1* | BP & CC | maintenance of postsynaptic specialization structure; modulation of chemical synaptic transmission; regulation of postsynaptic neurotransmitter receptor diffusion trapping; regulation of synapse disassembly; integral component of postsynaptic membrane; integral component of synaptic membrane | -1.21 | 0.001 |
| *Itpr3* | CC | integral component of presynaptic ER membrane | -1.17 | 0.023 |
| *Kcnj11* | BP & CC | voltage-gated ion channel activity involved in regulation of presynaptic membrane potential; integral component of presynaptic membrane | -1.13 | 0.050 |
| *Kcnj8* | BP & CC | voltage-gated ion channel activity involved in regulation of presynaptic membrane potential; integral component of presynaptic active zone membrane | 1.13 | 0.044 |
| *Nedd4* | BP & CC | regulation of postsynaptic neurotransmitter receptor endocytosis; regulation of protein catabolic process at postsynapse, modulating synaptic transmission; regulation protein catabolic process at postsynapse; postsynaptic cytosol | -1.46 | 0.020 |
| *Nr3c1* | CC | postsynaptic density, intracellular component | -1.24 | 0.018 |
| *Phb2* | CC | postsynaptic density; presynaptic active zone | 1.36 | 0.002 |
| *Plcb4* | BP & CC | modulation of chemical synaptic transmission; postsynaptic density; postsynaptic SER | -1.26 | 0.046 |
| *Plekhg5* | BP | regulation protein catabolic process at presynapse | 1.21 | 0.035 |
| *Ppp1r9a* | BP & CC | modulation of chemical synaptic transmission; postsynaptic actin cytoskeleton organization; postsynaptic actin cytoskeleton; postsynapse; postsynaptic density | -1.28 | 0.037 |
| *Pura* | BP & CC | dendritic transport of messenger ribonucleoprotein complex; postsynapse; postsynaptic density | -1.32 | 0.002 |
| *Rab11a* | BP & CC | neurotransmitter receptor transport, endosome to postsynaptic membrane; postsynaptic recycling endosome | 1.23 | 0.038 |
| *Rab3c* | CC | anchored component of synaptic vesicle membrane | -1.17 | 0.046 |
| *Rpl23* | BP & CC | translation at postsynapse; translation at presynapse; postsynaptic density; postsynaptic ribosome; presynaptic ribosome; synapse | 1.12 | 0.045 |
| *Rpl7a* | BP & CC | translation at postsynapse; translation at presynapse; postsynaptic density; postsynaptic ribosome; presynaptic ribosome; synapse | 1.54 | 0.049 |
| *Septin11* | BP & CC | regulation of synapse organization; postsynapse; postsynaptic specialization of symmetric synapse | -1.21 | 0.027 |
| *Snap23* | BP & CC | exocytic insertion of neurotransmitter receptor to postsynaptic membrane; postsynapse | -1.13 | 0.037 |
| *Snap47* | BP & CC | exocytic insertion of neurotransmitter receptor to postsynaptic membrane; postsynaptic density; postsynaptic active zone | 1.15 | 0.019 |
| *Snx27* | BP | regulation of postsynaptic membrane neurotransmitter receptor levels | -1.20 | 0.022 |
| *Snx9* | BP & CC | regulation of synaptic vesicle endocytosis; presynapse | -1.17 | 0.006 |
| *Sptbn1* | CC | postsynapse | -1.35 | 0.001 |
| *Stk38l* | BP | postsynapse organization | -1.16 | 0.031 |
| *Syn3* | BP & CC | synaptic vesicle clustering; synaptic vesicle cycle; extrinsic component of synaptic vesicle membrane | -1.41 | 0.039 |
| *Synj1* | BP & CC | regulation of postsynaptic neurotransmitter receptor endocytosis; synaptic vesicle uncoating; presynapse | -1.13 | 0.044 |
| *Tpd52* | CC | synapse | 1.17 | 0.047 |
| *Ube3a* | BP | modulation of chemical synaptic transmission | -1.18 | 0.028 |
| *Usp8* | BP & CC | regulation of protein catabolic process at postsynapse, modulating synaptic transmission; postsynaptic density | -1.36 | 0.026 |
| *Wasl* | BP & CC | postsynapse organization; postsynaptic actin cytoskeleton organization; regulation of postsynapse organization; postsynapse | -1.20 | 0.015 |

BP: biological process; CC: cellular component. Data are expressed as the fold changes of the level in six hours Hypox versus Norm (n=12 per group). Some genes are annotated by multiple categories.

**Table S6:** GSEA result using SynGO (P-value < 0.05)

| GO term ID | GO aspect | GO term name | GSEA P-value |
| --- | --- | --- | --- |
| GO:0050804 | BP | modulation of chemical synaptic transmission | 0.048 |
| GO:0099186 | BP | structural constituent of postsynapse | 0.023 |
| GO:0099572 | CC | postsynaptic specialization | 0.002 |
| GO:0014069 | CC | postsynaptic density | 0.010 |
| GO:0099092 | CC | postsynaptic density, intracellular component | 0.036 |

BP: biological process; CC: cellular component.

**Table S7:** Overview of differently regulated matrisome genes in hypoxia

| Group | Gene | Fold change | P-value |
| --- | --- | --- | --- |
| Collagens | *Col4a2* | 1.25 | 0.038 |
| Collagens | *Col4a1* | 1.34 | 0.029 |
| Collagens | *Col4a6* | -1.21 | 0.034 |
| ECM glycoproteins | *Vtn* | 1.26 | 0.033 |
| ECM glycoproteins | *Vwa1* | 1.13 | 0.045 |
| ECM glycoproteins | *Mfap1b* | -1.23 | 0.040 |
| ECM Regulators | *Fam20b* | -1.19 | 0.024 |
| ECM Regulators | *Adamts5* | -1.27 | 0.046 |
| ECM-affiliated Proteins | *Lgals3* | 1.49 | 0.002 |
| ECM-affiliated Proteins | *Sftpc* | 1.88 | 0.032 |
| ECM-affiliated Proteins | *Anxa8* | -1.19 | 0.045 |
| ECM-affiliated Proteins | *Gpc3* | -1.35 | 0.002 |
| ECM-affiliated Proteins | *Sema4d* | 1.18 | 0.019 |
| ECM-affiliated Proteins | *Sema5a* | -1.23 | 0.045 |
| ECM-affiliated Proteins | *Cd209d* | -1.16 | 0.034 |
| Proteoglycans | *Fmod* | 1.45 | 0.021 |
| Secreted Factors | *Cxcl12* | 1.45 | 0.049 |
| Secreted Factors | *Csf1* | -1.18 | 0.009 |
| Secreted Factors | *Igf1* | -1.19 | 0.011 |
| Secreted Factors | *Angptl4* | 1.60 | 0.012 |
| Secreted Factors | *Egf* | 1.18 | 0.026 |
| Secreted Factors | *Fgf13* | -1.28 | 0.030 |
| Secreted Factors | *Cntf* | -1.28 | 0.044 |

Data are expressed as the fold changes of the level in six hours Hypox versus Norm (n=12 per group).
